# Hierarchical Capillary Coating to Biofunctionlize Drug-Eluting Stent for Improving Endothelium Regeneration

**DOI:** 10.34133/2020/1458090

**Published:** 2020-08-20

**Authors:** Jing Wang, Yunfan Xue, Jun Liu, Mi Hu, He Zhang, Kefeng Ren, Yunbing Wang, Jian Ji

**Affiliations:** ^1^MOE Key Laboratory of Macromolecule Synthesis and Functionalization, Department of Polymer Science and Engineering, Zhejiang University, Hangzhou 310027, China; ^2^National Engineering Research Center for Biomaterials, Sichuan University, Chengdu 610064, China

## Abstract

The drug-eluting stent (DES) has become one of the most successful and important medical devices for coronary heart disease, but yet suffers from insufficient endothelial cell (EC) growth and intima repair, eventually leading to treatment failure. Although biomacromolecules such as vascular endothelial growth factor (VEGF) would be promising to promote the intima regeneration, combining hydrophilic and vulnerable biomacromolecules with hydrophobic drugs as well as preserving the bioactivity after harsh treatments pose a huge challenge. Here, we report on a design of hierarchical capillary coating, which composes a base solid region and a top microporous region for incorporating rapamycin and VEGF, respectively. The top spongy region can guarantee the efficient, safe, and controllable loading of VEGF up to 1 *μ*g/cm^2^ in 1 minute, providing a distinctive real-time loading capacity for saving the bioactivity. Based on this, we demonstrate that our rapamycin-VEGF hierarchical coating impressively promoted the competitive growth of endothelial cells over smooth muscle cells (ratio of EC/SMC~25) while relieving the adverse impact of rapamycin to ECs. We further conducted the real-time loading of VEGF on stents and demonstrate that the hierarchical combination of rapamycin and VEGF showed remarkable endothelium regeneration while maintaining a very low level of in-stent restenosis. This work paves an avenue for the combination of both hydrophobic and hydrophilic functional molecules, which should benefit the next generation of DES and may extend applications to diversified combination medical devices.

## 1. Introduction

Drug-eluting stent (DES) with controlled local release of antiproliferative drugs has become one of the most important medical devices in the treatment of coronary vascular diseases, saving millions of patients each year [1, 2]. Despite the excellent outcomes, extensive data from experimental and clinical trials reveal severe complications after DESs implantation including in-stent restenosis (ISR), neoatherosclerosis, and stent thrombosis (ST) [3–6]. In particular, the late and very late stent thrombosis (LAST) are highly lethal complications with incidence rate over 15% and mortality over 80% after >5-year implantation [7–11], which significantly impacts patients' life quality and costs huge medical resources. Recent clinical researches based on optical coherence tomography (OCT) have shown that the uncovered struts and neoatherosclerosis are most frequently observed in late/very late ST [12, 13]. The local release of antiproliferative drugs not only inhibits the migration and proliferation of smooth muscle cells (SMCs) but also impairs the recovery of the endothelium, leading to the uncovering of struts and delay of reendothelialization [14, 15]. Moreover, this dysfunction of endothelium and disturbed flow by uncovered struts contributes to the formation of neoatherosclerosis [16]. Therefore, it is of great interest to endow drug-eluting stents with endothelium regeneration property.

In nature, bioactive agents such as matrix proteins, nitric oxide, and growth factors play a central role in regulating cell behaviors and tissue regeneration [17–21]. For instance, vascular endothelial growth factor (VEGF) is a highly specific mitogen for vascular endothelial cells, which induces angiogenesis and regulates vascular growth and patterning [22–24]. We and other groups have previously demonstrated that the surfaces containing VEGF specifically promote adhesion and proliferation of endothelial cells [25–29]. In this context, introducing bioactive agents such as VEGF onto the surface of DES to promote EC proliferation and intima repair, without reducing antiproliferative capability on SMCs, would be very promising [30]. As simple as this concept sounds, combining aqueous-soluble and sensitive VEGF with organic-solvent-soluble drugs in the lipophilic polymer coating poses a great challenge. On one hand, physical adsorption of VEGF onto the surface would lead to uncontrolled burst release and very limited effects on ECs. On the other, direct encapsulation of VEGF within the drug coating would inevitably require contact with organic solvents, which significantly impairs the bioactivity of VEGF [31]. Moreover, current studies on coating methods have barely provided any insight into the harsh sterilization process of industrial production of DES, which further rises a challenge to VEGF bioactivity [32, 33]. Consequently, there remains an unmet need for coating design that enables the efficient combination of bioactive agents with drugs, particularly without impairing the bioactivity.

More recently, microporous materials with interconnected microstructures have aroused tremendous interest in drug delivery and tissue engineering [34–37]. This capillarity-based wicking process is simple, rapid, gentle, and generally independent of the solute in the adsorbed solution [38, 39], which would be favorable for the on-demand loading of biomacromolecules and fulfilling the “load-and-play” concept [40, 41]. We, therefore, suggest a hierarchical coating with dual-structured regions, which contains an underlying solid region for drug embedding and a top porous region for biomacromolecule loading, respectively. We envision that this hierarchical coating could realize the spatial combination of biomacromolecules with drugs and perform several distinguishing advantages: (1) biomacromolecules can be easily loaded via wicking action with highly controllable dosage and desirable bioactivities; (2) the loading process is simple and rapid, making it possible to perform just before the implantation and thus avoiding the harsh sterilizations; (3) the porous region could be utilized to modulate the release of underlying drugs.

**Scheme 1:**
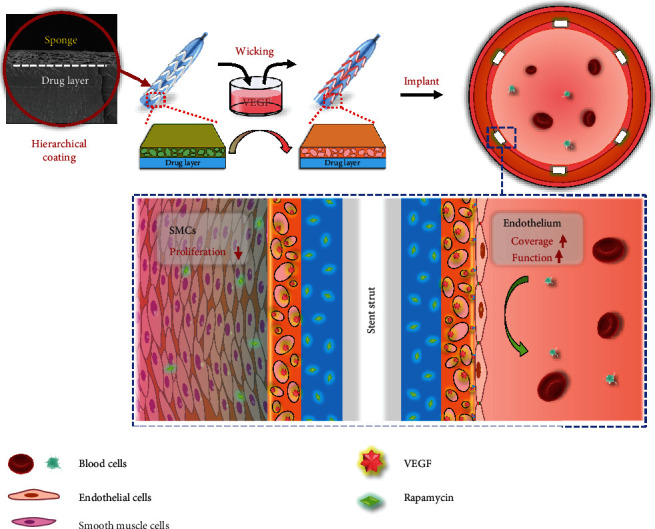
Schematic illustration of the hierarchical coating on the cardiovascular stent for regulating the intimal regeneration. The capillary-based wicking action could realize the distinctive real-time loading of VEGF during the surgery, which preserved the bioactivity of VEGF and impressively enhanced endothelium recovery while maintaining the inhibition of SMC proliferation after implantation.

Herein, we report a hierarchical coating that realizes the spatial combination of VEGF with rapamycin for promoting the regeneration of the endothelium after stent implantation (Scheme 1). Key features of this hierarchical coating include simple, efficient, and controllable loading of VEGF; fast loading process; and remarkable bioactivity. We verified the distinctive real-time loading capacity of the top-spongy layer via a simple wicking process. Then, we evaluated release profiles of VEGF and rapamycin and their effects on EC and SMC growth behaviors. We further studied the effects of the coating on EC gene expressions and m-TOR signaling. Last, the coating was prepared on the cardiovascular stent, and an *in vivo* experiment was carried out using a porcine coronary artery injury model. The endothelial regeneration, intimal repair, and in-stent restenosis were evaluated.

## 2. Results

### 2.1. Preparation of Hierarchical Spongy Coating

**Figure 1:**
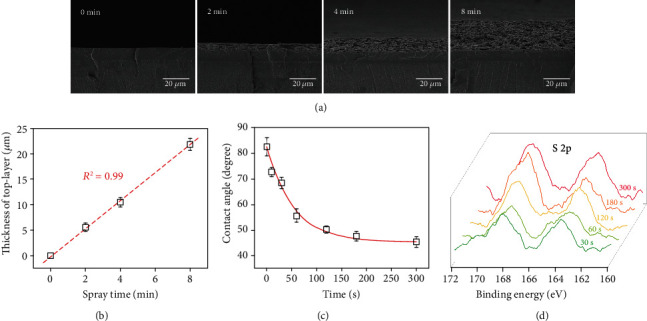
Preparation of the hierarchical coating. (a) SEM micrographs of the coating with different top layer spray time. (b) The thickness of the top layer as a function of spray time. Contact angle (c) and X-ray photoelectron spectrums (d) of the coating with different extent of heparinization based on UV irradiation.

The preparation of the hierarchical spongy coating is shown in Scheme S1. Briefly, the PLGA solution was first sprayed onto a substrate to form a solid layer, followed by spraying of PLGA-DA/PVP-mixed solution (mass ratio = 4 : 6) to form a top layer. The microporous spongy structure was then obtained by water leaching of PVP.  Figure 1(a) shows the cross-sectional morphology of the coating (after leaching) with different spraying time of top PLGA-DA/PVP. Clear dual structures with base solid and top porous regions could be observed. The thickness of the porous region can be easily controlled by spray time (Figure 1(b)). In order to increase surface wettability and affinity for VEGF, the top region was modified by heparin (Hep-SH) via thiol-ene “click” chemistry [42, 43].  Figure 1(c) shows the hydrophilicity of the coating with different extent of heparinization based on UV irradiation. The contact angle of the coating was decreased from 82.5 to 45.3 degrees after 5 min UV irradiation. Furthermore, FTIR (Figure S1, supporting information) and X-ray photoelectron spectrums (Figure 1(d)) show the decrease of the peak at ~1650 cm^−1^ and the increase of S2p peak, respectively. Collectively, we verified the successful heparinization of the coating. It should be noted that the heparin-conjugated coating dramatically reduced the protein adsorption and platelet adhesion, which significantly improved the blood compatibility (Figure S2, supporting information).

### 2.2. Spatial Combination of VEGF with Rapamycin

**Figure 2:**
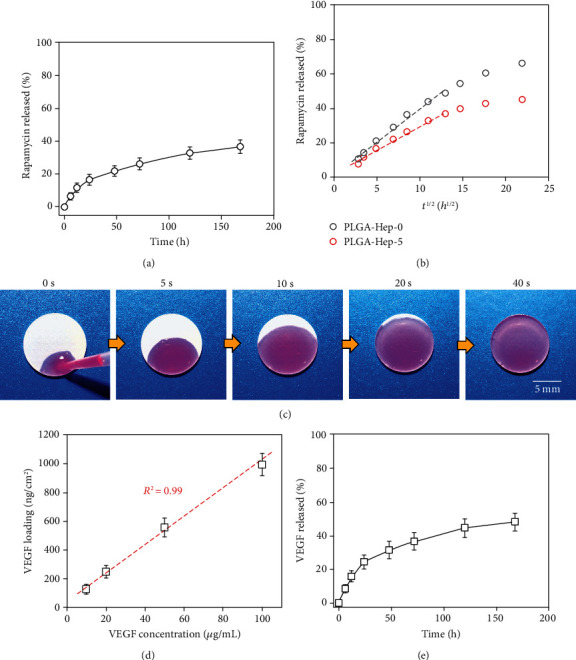
The loading and release profile of the hierarchical coating. (a) Rapamycin release profile as a function of time. (b) Comparison of rapamycin release with and without spongy top layer, respectively. (c) The wicking action of rhodamine B. (d) Loading of VEGF as a function of VEGF solution concentration. (e) VEGF release profile as a function of time.

Hydrophobic rapamycin was dissolved into PLGA solution and directly embedded into the base layer by spraying. Rapamycin density was set to 30 *μ*g/cm^2^ unless otherwise specified. The naked rapamycin coating did not support ECs and SMCs adhesion due to the initial burst release of the drug (Figure S3, supporting information). As shown in Figure 2(a), for the coating with a 5 *μ*m microporous top layer, an initial burst release with 11.8 ± 2.8% of total loading was observed at the first 12 h. The cumulative release of rapamycin reached 36.7 ± 4.1% at 7 d. Moreover, we verified that the microporous top layer reduced rapamycin release. By fitting release percentage against the square root of release time (Figure 2(b)), the release profiles at the early stage followed a Fickian diffusion mechanism [34, 44, 45]. According to Higuchi's equation, the theoretical release profile can be described as follows:

(1) $$\begin{array}{c} f\left(t\right)=4{\left(\frac{Dt}{\pi x^{2}}\right)}^{0.5}, \end{array}$$

where *f*(*t*) is the drug release at time *t*, *x* is the thickness of the coating, and *D* is the diffusion coefficient. Thus, diffusion coefficients of the naked rapamycin-PLGA coating and the dual-structured coating were 2.6 × 10^−12^ and 1.9 × 10^−12^ cm^2^/s, respectively. Since the thickness of the top microporous layer could be precisely controlled, it provides a potential way to modulate the rapamycin release [46, 47].

Then, the loading capacity of the top spongy region was evaluated. As shown in Figure 2(c), we first used rhodamine B (RB) as an indicator to demonstrate the loading process. Once a drop of RB solution contacted the coating, the pink liquid was rapidly adsorbed into the coating within seconds. We also employed the FITC-labeled poly (L-lysine) (PLL-FITC) to verify a very good distribution of biomacromolecule after the loading process (Figure S4, Supporting information).  Figure 2(d) shows the VEGF loading densities as a function of VEGF solution concentrations. The density of VEGF showed a linear relationship against the solution concentration, suggesting a highly controllable VEGF loading dosage. Besides, it should be notable that the loading density reached up to 1 *μ*g/cm^2^ within only 1 min (solution concentration was 100 *μ*g/mL), while such high VEGF density usually took tens of minutes to hours by other methods [28]. The VEGF loading efficiency showed a similar level (ranging from 32% to 26%) at different solution concentrations (Figure S5, Supporting information), indicating a reliable VEGF loading process. More importantly, the incorporated VEGF showed considerable activity due to this mild and ultrafast wicking process (Figure S6, Supporting information). We further measured the VEGF release profile. As shown in Figure 2(e), the loading VEGF showed a rapid release of 24.4 ± 4.1% at the first 24 h and a total 48.1 ± 5.2% of VEGF at 72 h. The top spongy layer on one hand performed as a diffusion barrier that contributed to the prolonged release of rapamycin and, on the other hand, provided a biomimetic binding site for the sustained release of VEGF [48, 49].

It should be noted that the release behavior of VEGF and rapamycin both showed a burst release at the initial 24 h and a sustained diffusion-controlled release without an obvious degradation-induced acceleration. To verify the degradation-accelerated release behavior, we employed lipase in the release medium at a concentration of 1 mg/mL according to our previous report (Figure S7, Supporting information) [50]. The total release of VEGF and rapamycin at 7 d was 59.4% and 42.1%, respectively. Then, the cumulative release of both VEGF and rapamycin was notably accelerated with prolonged time. The total release of VEGF and rapamycin at 14 d was 89.8% and 68.3%, respectively. The SEM micrographs of hierarchical coatings before and after 14-day release confirmed the degradation of PLGA that contributed to the accelerated release behavior.

### 2.3. Coculture of ECs with SMCs In Vitro

**Figure 3:**
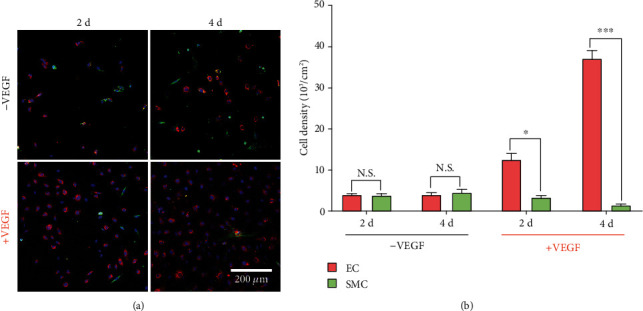
Coculture of ECs with SMCs. Confocal micrographs (a) and corresponding cell density (b) of ECs and SMCs on the hierarchical coatings (*n* = 5, ^∗^*P* < 0.05, ^∗∗∗^*P* < 0.001).

To verify the effects of our coating on ECs and SMCs, cell proliferation was first measured. We studied the proliferation behavior of SMCs and ECs separately (Figure S8, supporting information) and confirmed that the spatial combination of VEGF with rapamycin selectively promoted the growth of ECs. Then, the coculture test was performed as shown in Figure 3. For the coating without loading VEGF, both EC and SMC proliferations were dramatically inhibited with poorly spread morphology. In contrast, on the VEGF loaded coating, the proliferation of ECs was significantly enhanced when the proliferation of SMCs remained inhibited (Figure 3(a)). The cell density ratio of ECs/SMCs reached as high as 25 by the combination of VEGF with rapamycin (Figure 3(b)). However, incorporating VEGF without rapamycin promoted the proliferation of ECs but showed little effect on inhibiting SMCs (Figure S9, supporting information), confirming that the promotion strategy regardless of SMCs was insufficient to obtain a pure endothelium [26, 51]. Therefore, the spatial combination of VEGF with rapamycin by hierarchical coating suppressed the undesired proliferation of SMCs and promoted the favorable growth of ECs, which should be beneficial for the endothelialization on stents.

### 2.4. Effect of the Hierarchical Coating on the Regulation of ECs

**Figure 4:**
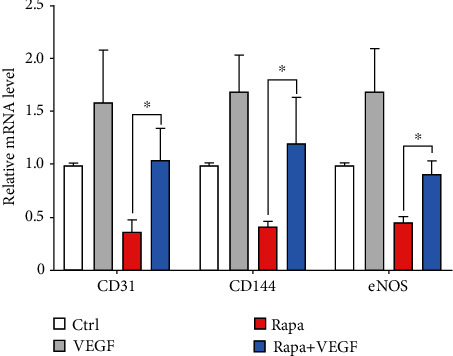
Relative expression of endothelial function related genes. Values normalized to control samples (*n* = 3, ^∗^*P* < 0.05).

We next investigated the effect of hierarchical coatings on the expression of endothelial function-related genes. Three representative endothelial function-related genes were adopted, in which the PECAM-1/CD31 and VE-cadherin/CD144 are important cell junction components between ECs for maintaining the integrity of endothelium, and eNOS plays an important role in the generation of NO [52–54]. As shown in Figure 4, the incorporation of VEGF remarkably enhanced the expression of endothelial function-related genes as compared to the rapamycin coatings. We also confirmed that the incorporation of VEGF could partially relieve the impact of rapamycin on the NO generation of ECs (Figure S10, supporting information). Our previous researches have revealed that bound matrix of growth factor can selectively promote the adhesion and proliferation of ECs [26, 29]. However, the regeneration of endothelium not only requires the purified coverage of endothelial cells but also demands the recovery of endothelium functions. Our results, for the first time, suggested that the VEGF was able to favor the improvement of endothelial function after the severe impact of rapamycin, which would be vital to the endothelium regeneration in clinical applications.

**Figure 5:**
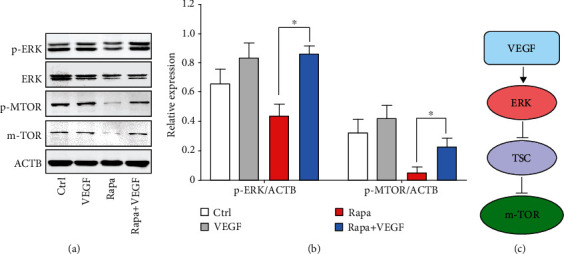
Regulation of the m-TOR signaling in ECs via the spatial combination of VEGF with rapamycin. (a) Western blot for ERK1/2 and its downstream effector m-TOR. (b) Quantification of Western blot bands. Values normalized to ACTB. (c) Potential signaling pathway related to the regulation of m-TOR by VEGF (*n* = 3, ^∗^*P* < 0.05).

Then, we evaluated the regulation of m-TOR signaling within ECs because it is well accepted that m-TOR is the direct target of rapamycin [1, 3, 55]. As shown in Figures 5(a) and 5(b), the phosphorylation level of m-TOR was significantly depressed when the coating contained rapamycin only, which could be attributed to the binding of rapamycin with FK-binding protein 12 [1, 56]. The phosphorylation level of ERK 1/2 was also downregulated. Previous works have revealed that m-TOR plays a central role in regulating the production of protein, lipids, and nucleotides and controls the balance between anabolism and catabolism [57]. The high concentration of rapamycin significantly inhibited m-TOR, thus leading to the disorder of autophagy, which might contribute to the downregulation of ERK 1/2. In contrast, the incorporation of VEGF dramatically enhanced the phosphorylation level of ERK 1/2, suggesting that the bioactivity of VEGF was properly preserved during the wicking action [24, 28]. Moreover, the m-TOR level was dramatically enhanced (>4-fold), indicating that the incorporation of VEGF can assuage the impact of rapamycin. Studies have revealed that m-TOR is a downstream mediator of several growth factor-dependent signaling pathways via the inhibition of Tuberous Sclerosis Complex (TSC). In particular, receptor tyrosine kinase-dependent Ras activates the MAP kinase ERK, which contributes to the inhibition of TSC and activation of m-TOR [57, 58]. Our findings verify this signaling pathway (Figure 5(c)) and demonstrate that efficient delivery of VEGF is a valid way to relieve the side effect of rapamycin to ECs. We further found that the spatial combination of VEGF with rapamycin significantly reduced the apoptosis rate as compared to the rapamycin coating (Figure S11, supporting information), indicating that the loading of VEGF was effective to preserve the activity of ECs.

### 2.5. In Vivo Evaluation of Hierarchical Coating on Stents

**Figure 6:**
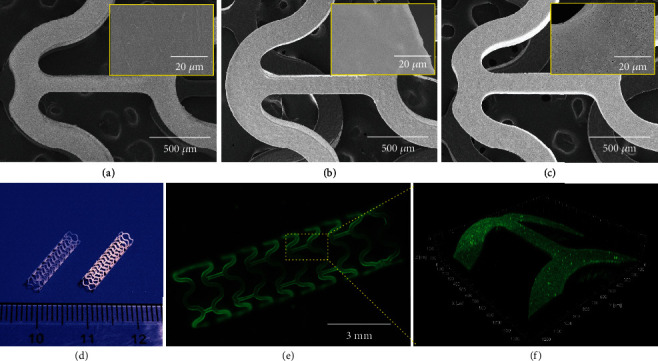
Construction of hierarchical coating onto the stents: SEM micrographs of BSs (a), RESs (b), and VRSs (c). (d) Digital photo of BSs (left) and VRSs (right). Fluorescence micrographs (e) and confocal scanning (f) of VRSs after loading of PLL-FITC.

Before the *in vivo* evaluation, we prepared the hierarchical coatings onto the PLLA biodegradable stents. Three types of stents were prepared, including bare stents (BSs) as control, rapamycin-coated stents (RESs), and bilayer-coated stents as VEGF/rapamycin combination stents (VRSs). As shown in Figures 6(a)–6(c), all these stents presented smooth surface morphology, in which RESs and VRSs did not show any peeling of the coatings. The VRSs showed obvious white opaque appearance as compared to the BSs (Figure 6(d)), which could be attributed to the light scattering effect of spongy microstructure. The loading capacity of VRSs was evaluated by using PLL-FITC as model biomacromolecule (Figures 6(e) and 6(f)). The green fluorescence signal was uniformly distributed on the struts, indicating that PLL-FITC was evenly loaded within the coating via wicking action.

**Figure 7:**
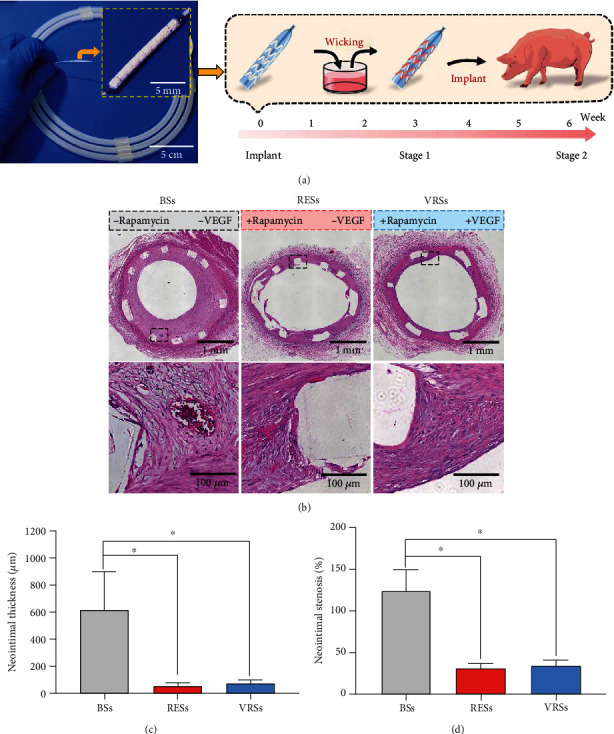
Histological analysis results at 6 weeks. (a) The digital photos of the stent with a hierarchical coating (left) and the typical wicking process during the surgery (right). (b) The micrographs of the H&E-stained cross-section slices of the arteries with BSs, RESs, and VRSs, respectively. Histological analysis of neointimal thickness (c), and the percentage of neointimal stenosis (d) (*n* = 3, ^∗^*P* < 0.05).

Then, we conducted in vivo animal experiments using a porcine coronary artery injury model.  Figure 7(a) shows the typical VEGF wicking process just before the surgery, which represented the key feature of our coating design that insures the bioactivity of VEGF. After 42 days (6 weeks) implantation, coronary angiography showed that the BSs generated a significant lumen loss, while the RESs and VRSs showed no significant difference (Figure S12, supporting information). Correspondingly, the histomorphometric analysis showed that RESs and VRSs dramatically reduced the intima hyperplasia as compared to BSs. The average neointimal thickness in RESs and VRSs was reduced by 91.4% and 87.7%, respectively (Figure 7(c)). Meanwhile, the neointimal stenosis rate of RESs and VRSs was also 74.8% and 72.8% lower than that of BSs, respectively. Furthermore, the stent surface of VRSs was covered with intact intima compared to the partial coverage in the RESs, suggesting that VEGF loaded by wicking action successfully relieved the impact of rapamycin and promoted the stent endothelialization.

**Figure 8:**
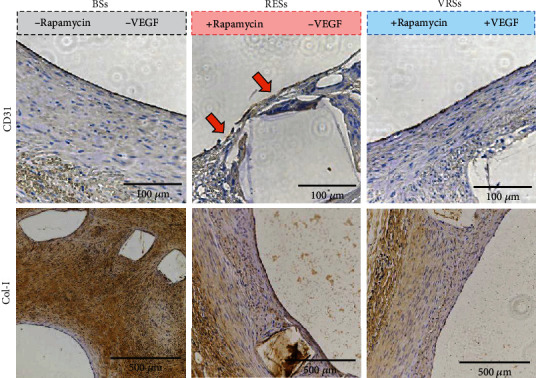
Immunohistochemical analysis of the stented arterial segments for CD31 and Col-I, respectively.

To evaluate the antihyperplasia of SMCs and the recovery of the endothelium, immunohistochemical staining for Col-I and CD31 was performed (Figure 8). Compared to the BSs, both RESs and VRSs showed significantly depressed expression of Col-I, verifying that the eluting of rapamycin is essential for inhibiting excessive expression of SMC-related extracellular matrix. Moreover, the neointima on RESs showed partial coverage of CD31, indicating the delayed repair of the endothelium. In contrast, VRSs showed the intact coverage of CD31-positive ECs, further proving the better regeneration of endothelium.

**Figure 9:**
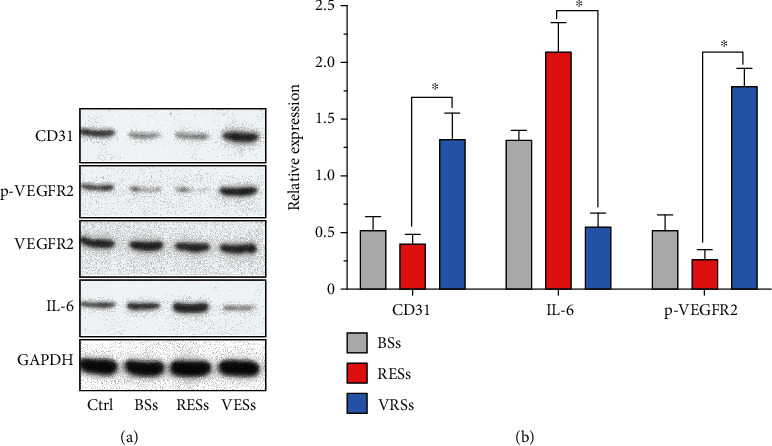
(a) Western blot analysis of arterial segments implanted with BSs, RESs, and VRSs, respectively. Ctrl represents the native arterial segments. (b) Quantification of Western blot bands. Values normalized to GAPDH and then normalized to Ctrl (*n* = 3, ^∗^*P* < 0.05).

To verify the effect of the spatial combination of VEGF with rapamycin by hierarchical spongy coating, we evaluated the protein expression level that related to the regeneration process after implantation for 21 days (3 weeks). As shown in Figures 9(a) and 9(b), the phosphorylation level of VEGFR2 in VRSs was over 6-fold higher than RESs, suggesting that the VEGF loaded by wicking action maintained the bioactivity. Moreover, the expression of CD31, a specific functional protein of ECs [51, 54], was downregulated in the arteries RESs but remarkably increased in the arteries of VRSs (~225% high than the RESs), indicating that the VEGF-incorporated stents showed better recovery of endothelium function. In addition, we also measured the expression level of IL-6, an important type of inflammation-associated protein [51, 59], to evaluate inflammation after implantation. The VRSs significantly reduced the expression of IL-6 level as compared to the BSs (~58% decrease) and RESs (~73% decrease), probably due to the promoted reendothelialization.

All these results demonstrated that both RESs and VRSs showed reliable antirestenosis property, in which the VRSs with the effective delivery of VEGF performed better regeneration of the endothelium. Together with the antirestenosis property and better reendothelialization, our hierarchical coating proposed a reliable way to promote the regeneration of the endothelium, validating the potentials in clinical applications.

## 3. Discussion

We have developed a hierarchical capillary coating, composed by a dense rapamycin-loaded layer and a spongy top layer for incorporating VEGF, as an effective approach to inhibit in-stent restenosis while promoting the regeneration of endothelium after stent implantation. The heparin conjugation to the spongy top layer endowed the coating ultrafast and controllable loading of VEGF up to 1 *μ*g/cm^2^, ensuring the distinctive real-time loading to properly preserve the bioactivity. Moreover, we demonstrated that the spatial combination of VEGF with rapamycin not only promoted the competitive growth of ECs over SMCs but also relieved the severe impact of rapamycin on the endothelial function, presumably due to the upregulation of inhibited m-TOR signaling. Based on this, we coated the biodegradable stents with our bilayer coating, carried out the standard sterilization procedure, and performed a “load-and-play” concept to incorporate VEGF by a wicking process during the surgery. We verified that the bioactivity of VEGF was considerably maintained, which, by cooperating with rapamycin, realized the sufficient depression of in-stent restenosis and efficient regeneration of endothelium. Therefore, we believed that this approach paves an avenue for the combination of vulnerable bioactive agents with conventional systems and may facilitate new applications of bioinspired materials.

## 4. Materials and Methods

### 4.1. Materials

2-Hydroxy-4′-(2-hydroxyethoxy)-2-methylpropiophenone (I2959), heparin, FITC-labeled poly-L-lysine (PLL-FITC, Mw 30-70 kDa), polyvinylpyrrolidone (PVP, Mw ~40 kDa), rhodamine B, 1-ethyl-3-(3-dimethylaminopropyl)carbodiimide (EDC), N-hydroxysulfosuccinimide (NHSS), and cystamine dihydrochloride, heparin, 1,4-dithiothreitol (DTT), lipase, and rapamycin were purchased from Sigma-Aldrich. PBS was purchased from Sango Biotech (Shanghai, China). Poly(D,L-lactide-co-glycolide) (PLGA, LA : GA = 75 : 25, Mn ~65 kDa) and PLGA diacrylate (PLGA-DA, LA : GA = 75 : 25, Mn ~65 kDa) were purchased from Jinan Daigang Biomaterial Co., Ltd. (Jinan, China). Annexin V-FITC/PI kit was purchased from Thermo Fisher Scientific Inc. Recombinant human VEGF165 was purchased from PeproTech (Rocky Hill, USA). The deionized (DI) water (>18 M*Ω* cm) used in all experiments was provided by a Millipore Milli-Q water purification system. All materials were used as received without further purification.

### 4.2. Synthesis of Thiolated Heparin (Heparin-SH)

Thiolated heparin was obtained according to a previously reported procedure [41, 59]. Briefly, 10 mL heparin solution (20 mg/mL in water) was firstly treated with EDC (1.5 mmol) and NHSS (1.5 mmol) for 2 h to activate the carboxyl groups, followed by reaction with cystamine dihydrochloride (1.5 mmol) overnight at room temperature to conjugate cystamine onto heparin (Heparin-Cys). Then, the reaction mixture was exhaustively dialyzed against deionized water and treated with a 5-fold excess of DTT to cleave the disulfide bonds. The thiolated heparin (Heparin-SH) was further dialyzed against deionized water for 2 d with the addition of HCl (0.1 M) to adjust the pH to ~5.0 for the protection of the thiol group. Finally, the purified Heparin-SH was lyophilized and stored at -20°C until use. The thiolation was verified by ^1^H-NMR in D_2_O.

### 4.3. Construction of the Hierarchical Coatings

The sequent spray was performed using PLGA solution as the base layer and PLGA-DA/PVP-mixed solution (mass ratio 4/6) as the top layer, respectively. For the preparation of the rapamycin-loaded base layer, rapamycin was dissolved into PLGA solution at a concentration of 0.77 mg/mL before sequent spray. Then, the coating was immersed into the heparin-SH solution for leaching, followed by UV irradiation for different time (80 mW/cm^2^, Intelli-Ray 400, USA). After the UV treatment, the coating was rinsed with Milli-Q water several times, blown dry, and kept at 4°C before use. The morphology of coating was observed by SEM (Hitachi S4800, Japan); The coating hydrophilicity was determined by a contact angle test using 2 *μ*L water droplets; the photos were taken 10 s after contact with spongy films (DSA100, Germany); the atomic composition of the coatings was obtained by XPS (VG ESCALAB MARK II, UK).

### 4.4. Blood Compatibility Analysis

The fresh rabbit blood was centrifuged at 1500 rpm for 15 min to obtain platelet-rich plasma. Samples were placed in 24-well plates and incubated with 0.5 mL platelet-rich plasma for 2 h at 37°C. Afterward, the platelet-rich plasma treated samples were gently rinsed with PBS 3 times and fixed by treatment with 2.5% glutaraldehyde for 30 min. All samples were dehydrated by graded ethanol for 10 min each (20%, 40%, 50%, 60%, 70%, 80%, 90%, and 100%) and then observed by SEM (Hitachi S4800, Japan).

The fresh rabbit blood was centrifuged at 3000 rpm for 10 min to obtain platelet-poor plasma (PPP). All samples were immersed in PPP and fibrinogen solution (1 mg/mL in PBS) in 24-well plates, respectively. After 2 h treatment in 37°C condition, the samples were rinsed with PBS for 3 times and incubated with 0.5% Triton X-100 solution for further 12 h with shaking. BCA kit was applied to determine the total protein adsorption on the sample. APTT/TT tests were performed by incubation with platelet-poor plasma and activated partial thromboplastin time/thrombin time (APTT/TT) reagents (Jiancheng Bioengineering, China) successively. The clotting time was recorded. Each group contains at least 3 parallel samples.

The whole blood clotting time (WBCT) test was performed using fresh rabbit (adding 10 vol% 0.2 M CaCl_2_ before the test). Different samples were treated with whole blood for various times (5, 10, and 15 min), then gently rinsed with PBS. Digital photos were taken and analyzed by ImageJ software (NIH, Bethesda, USA).

### 4.5. Loading and Release of Rapamycin and VEGF

The release profile of rapamycin was performed in the release medium (5% DMSO +95% pH 7.4 PBS). Samples were taken out at different periods, rinsed with pure water, and vacuum dried at 37%, then dissolved by ethyl acetate and measured by a UV-vis spectrophotometer (UV-2550, Shimadzu, Japan). For the lipase-accelerated release, the lipase was added into the release medium at a concentration of 1 mg/mL.

For the loading of VEGF, samples were placed in a 24-well plate with the addition of 50 *μ*L VEGF solution for 1 min wicking action. Then, samples were rinsed by BSA solution (0.2 mg/mL in PBS) for 3 times. The loading dosage of VEGF was obtained by a subtraction of total VEGF in the wicking solution and residual VEGF in the BSA solution. The release behavior of VEGF was performed in the release medium (0.2 mg/ml BSA in PBS). The release solution was frequently replaced with a fresh BSA solution at supposed time points. VEGF ELISA kit was applied to determine the content of VEGF in various solutions. For the lipase-accelerated release, the lipase was added into the release medium at a concentration of 1 mg/mL.

VEGFR2 internalization analysis was performed to study the bioactivity of VEGF as the previous report [60]. Parts of the samples were sterilized and set as the control. The coatings were placed in 24-well plates and immersed into EC culture medium without serum and VEGF for 12 h. HUVECs seeded on the glass slides were starved for 2 h in serum-free medium, followed by incubation in the collected solution for 30 min on ice. After incubation, the cells were cultured in a VEGF-free medium for further 3 h. Then, all samples were gently washed with PBS, fixed with 4% paraformaldehyde, and treated with 0.1% Triton X-100. Cell staining was performed for nuclei (1 : 100, DAPI, Sigma-Aldrich), F-actin (1 : 500, rhodamine phalloidin, Sigma-Aldrich), and VEGFR2 (1 : 200, monoclonal anti-VEGFR2 antibody, Sigma-Aldrich). All samples were fixed onto clean coverslips with the antifade reagent. The VEGFR2 internalization was observed by fluorescence microscopy (DS-Ri2, Nikon, Japan).

The NO generation of ECs was detected using a NO fluorescence probe (Beyotime, China) according to our previous work [61]. Briefly, the rapamycin-coated samples and rapamycin/VEGF-loaded samples were immersed in cell culture medium in 24-well plates for 24 h; the extraction culture medium of different samples was collected. ECs were cultured at a density of 50000/well in 24-well plates for 12 h; then the culture medium was replaced by the extraction culture medium. After 12 h culture, the cells were washed with PBS and treated with a NO fluorescence probe (in phenol red free medium) for 20 min, followed by PBS washing and paraformaldehyde fixation. The NO generation was observed fluorescence microscopy (DS-Ri2, Nikon, Japan), and the results were analyzed using ImageJ software (NIH, Bethesda, USA).

### 4.6. Coculture

ECs and SMCs were seeded on different samples at a density of 5000 cells per well (total of 10000 cells per well). The endothelial cell medium (ECM) was used in coculture for different culture times. Then, all samples were gently washed with PBS, fixed with 4% paraformaldehyde, and treated with 0.1% Triton X-100. Cell staining was performed for nuclei (DAPI, Sigma), von Willebrand factor (anti-vWF-red, Invitrogen), and *α*-smooth muscle actin (anti-*α*-SMA-green, Sigma-Aldrich). All samples were fixed onto clean coverslips with the antifade reagent and observed by confocal microscopy (FV3000, Olympus, Japan).

### 4.7. Evaluation of m-TOR Signaling and Endothelial Function-Related Genes in ECs

Different samples were placed into 6-well plates. ECs were seeded at a density of 1 × 10^5^ cells per well and cultured for 48 h. Then, the cells on different surfaces were washed gently with PBS and lysed for protein isolation. A Western blot assay was employed to study the m-TOR signaling, including the phosphorylation of ERK1/2 and m-TOR.

The expression of platelet endothelial cell adhesion molecule-1 (PECAM-1/CD31), VE-cadherin/CD144, and endothelial nitric oxide synthase (eNOS) were analyzed by real-time quantitative PCR (RT-qPCR) assay (CFX384, BioRad, Hercules, CA, USA). ECs were on different samples at a desired density, then lysed for RNA extraction by TRIzol reagent (Haogene Biotech, Shanghai, China). The primer sequences for tested genes are shown in supporting information.

### 4.8. Cell Viability

HUVECs were seeded onto the hierarchical spongy coatings (rapamycin density: 30 *μ*g/cm^2^) with and without incorporation of VEGF. TCPS was used as the control. After 24 h incubation, cells were harvested and treated with Annexin V-FITC/PI kit, then detected by flow cytometry (BD FACSCalibur, USA).

### 4.9. Stent Implantation and Stent Segment Analysis

Biodegradable stents (3 mm in diameter, 13 mm in length) without drug coating were provided by Xing Tai Pu Le Biomedical Technology Co., Ltd. Hierarchical coating was constructed on to the stent, containing 50 ± 3 *μ*g of rapamycin. Bare stents were used as control. For the VEGF content, 50 *μ*L VEGF solution (100 *μ*g/mL in pure water) was carefully added onto the stent in the Eppendorf Lobind tube. After the wicking action for 1 min, the stent was washed by BSA solution (0.2 mg/mL in PBS) for 3 times, the rinsing solution was collected. The total VEGF loading content was 0.82 ± 0.12 *μ*g/stent, which was determined by the subtraction of the total VEGF in the wicking solution and residual VEGF in BSA solution using VEGF ELISA kit.

The loading of VEGF was performed right before implantation via wicking in VEGF solution (100 *μ*g/mL) for 1 min, followed by a quick wash using normal saline. All of the animal experiments were performed by the Gateway Medical Innovation Center in Shanghai. Ten white minipigs (25-35 kg) with a total of 20 stents were used in this study following the guidelines of the Chinese Animal Care and Use Committee standards. Two evaluation stages with different time points (21 d and 42 d) were performed with 3 bare stents, 3 rapamycin stents, and 4 Rapa/VEGF stents for each stage group. It was ensured that no branches were presented in the stented region. Digital angiography was recorded before and after implantation. All animals were treated with gentamicin sulfate (5 mg/kg) by intramuscular injection in the next three days and fed a normal diet containing 150 mg aspirin and 300 mg clopidogrel every day.

Pigs were euthanized at 21 d and 42 d. The stented artery samples with implantation for 21 d were excised and washed quickly with saline, followed by freezing in liquid nitrogen. The frozen samples were homogenized in RIPA lysis buffer with protease inhibitors. Western blot assay was applied to evaluate the expression of CD31, IL-6, and VEGF receptor-2, respectively. The healthy arteries without stent implantation were adopted as the control.

The stented artery samples with implantation for 42 d were excised and fixed in 5% phosphate-buffered formalin for 24 h. Then, samples were embedded in paraffin for cross-section slicing (3 representative sections for each artery segment). Four slices were prepared at each section, one of which was stained by hematoxylin-eosin (HE) (PharmaLegacy Co., Ltd. China). Histological analysis of the neointimal hyperplasia was performed according to a previous report [6, 50]. The rest three of the slides were processed for immunohistochemistry of CD31, *α*-smooth muscle actin (*α*-SMA), and Col-I, respectively. Representative micrographs were taken by an optical microscope (DS-Ri2, Nikon, Japan).

### 4.10. Statistical Analysis

All data were obtained from at least three independent experiments with at least three parallel samples per condition in each experiment and expressed as the mean ± standard deviation (SD). Statistical significance was assessed with Student's *t*-test, and a probability value of *P* < 0.05 is considered as statistically significant.

## Supplementary Materials

Supplementary Materials Table S1: primer sequences and PCR product lengths. Scheme S1: schematic illustration of the preparation of the hierarchical coating. The capillarity based wicking action realizes the spatial combination of VEGF with drugs. Figure S1: heparin conjugation to the hierarchical coatings: (a) 1H-NMR spectrum of heparin-SH and (b) FTIR spectrum of the hierarchical coating before and after heparin conjugation, respectively. The peak decrease at 1640 cm^−1^ indicates the reaction between the methacrylate group and the thiol group. Figure S2: blood compatibility of hierarchical coating before and after heparin conjugation: (a) total protein adsorption by immersion in platelet-poor plasma and fibrinogen solution (1 mg/mL in PBS) for 2 h, respectively, (b) platelet adhesion on the coatings, (c) APTT and TT tests, and (d) whole blood test at different time points. The right image shows histogram analysis of the samples of 15 min whole blood treatment (*n* = 3, ^∗^*P* < 0.05). Figure S3 the adhesion and proliferation of ECs and SMCs on the hierarchical coatings with different thicknesses of the top spongy region. With the increased thickness of top spongy layer, both ECs and SMCs showed better growth behavior. Figure S4: confocal fluorescence micrographs with 3d view (a) and side view (b) showing the distribution of PLL-FITC within the hierarchical coating. Figure S5: VEGF loading efficiency at different solution concentrations. The VEGF loading efficiency showed a similar level at different solution concentrations, indicating a reliable VEGF loading process. Figure S6: the internalization of VEGFR2 within ECs. The sterilized coating showed little effect on the VEGFR2 internalization, indicating the loss of bioactivity. In contrast, VEGF loaded by wicking action promoted the VEGFR2 internalization (red spot). Figure S7: the lipase-accelerated release behavior of hierarchical coating: (a) cumulative release of VEGF and rapamycin in 14 d. (b) SEM micrographs of hierarchical coating before and after 14-day release. Rapa-s1: PLGA-DA/PVA~0.4; Rapa-s2: PLGA-DA/PVP~0.6. Figure S8: the cell growth behavior of ECs and SMCs on the hierarchical coatings before and after VEGF incorporation: (a) fluorescence micrographs of ECs on the hierarchical coatings with various VEGF density. The VEGF density of V-20, V-50, and V-100 was 0.25, 0.55, and 1.01 *μ*g/cm^2^, respectively; (b) fluorescence micrographs of the cells (blue: DAPI; green: F-actin); (c) corresponding cell density of the ECs and SMCs (*n* = 5, ^∗∗∗^*P* < 0.001). Figure S9: coculture of ECs with SMCs on rapamycin-free coatings. Confocal micrographs (a) and corresponding cell density of ECs and SMCs on the hierarchical coatings with/without VEGF loading, respectively (*n* = 5, ^∗^*P* < 0.05, ^∗∗∗^*P* < 0.001). Figure S10: the nitric oxide (NO) generation of ECs: (a) fluorescence micrographs of ECs after treatment with different samples; the red NO fluorescence probe is used to detect the NO generation. The cell density of ECs (b) and relative fluorescence intensity (c) after treatment with different samples (the relative fluorescence intensity was normalized against control; *n* = 5, ^∗∗∗^*P* < 0.001). Figure S11: evaluation of the cell apoptosis by flow cytometry. The coatings containing rapamycin alone induced substantial apoptosis of the ECs, while the spatial combination of VEGF with rapamycin dramatically enhanced cell viability. Figure S12: coronary angiography after implantation for 6 weeks. The bare stents showed obvious in-stent restenosis, while the rapamycin-coated stents and VEGF/rapamycin-cocoated stents remained patency.
