# Supplementary material for: Hierarchical Capillary Coating to Biofunctionlize Drug-Eluting Stent for Improving Endothelium Regeneration
Source: Research (Wash D C). 2020 Aug 20;2020:1458090. doi: 10.34133/2020/1458090 (PMC7455884; doi:10.34133/2020/1458090)
Supplement: Supplementary Materials — Table S1: primer sequences and PCR product lengths. Scheme S1: schematic illustration of the preparation of the hierarchical coating. The capillarity based wicking action realizes the spatial combination of VEGF with drugs. Figure S1: heparin conjugation to the hierarchical coatings: (a) 1H-NMR spectrum of heparin-SH and (b) FTIR spectrum of the hierarchical coating before and after heparin conjugation, respectively. The peak decrease at 1640 cm−1 indicates the reaction between the methacrylate group and the thiol group. Figure S2: blood compatibility of hierarchical coating before and after heparin conjugation: (a) total protein adsorption by immersion in platelet-poor plasma and fibrinogen solution (1 mg/mL in PBS) for 2 h, respectively, (b) platelet adhesion on the coatings, (c) APTT and TT tests, and (d) whole blood test at different time points. The right image shows histogram analysis of the samples of 15 min whole blood treatment (n = 3, ∗P < 0.05). Figure S3 the adhesion and proliferation of ECs and SMCs on the hierarchical coatings with different thicknesses of the top spongy region. With the increased thickness of top spongy layer, both ECs and SMCs showed better growth behavior. Figure S4: confocal fluorescence micrographs with 3d view (a) and side view (b) showing the distribution of PLL-FITC within the hierarchical coating. Figure S5: VEGF loading efficiency at different solution concentrations. The VEGF loading efficiency showed a similar level at different solution concentrations, indicating a reliable VEGF loading process. Figure S6: the internalization of VEGFR2 within ECs. The sterilized coating showed little effect on the VEGFR2 internalization, indicating the loss of bioactivity. In contrast, VEGF loaded by wicking action promoted the VEGFR2 internalization (red spot). Figure S7: the lipase-accelerated release behavior of hierarchical coating: (a) cumulative release of VEGF and rapamycin in 14 d. (b) SEM micrographs of hierarchical coating before [file 1458090.f1.docx]

A Hierarchical Spongy Coating to Combine VEGF and Rapamycin for Improving Intima Repair on Drug-eluting Stent

Jing Wang^1^, Yunfan Xue^1^, Jun Liu^1^, Mi Hu^1^, He Zhang^1^, Kefeng Ren^1,^*, Yunbing Wang^2^ and Jian Ji^1,^*

^1^ MOE Key Laboratory of Macromolecule Synthesis and Functionalization, Department of Polymer Science and Engineering, Zhejiang University, Hangzhou 310027, P.R. China
^2^ National Engineering Research Center for Biomaterials, Sichuan University, Chengdu 610064, P.R. China

^*^Corresponding author: Prof. Kefeng Ren, Prof. Jian Ji

***Table S1.*** *Primer sequences and PCR product lengths.*

| Gene | Primer Sequences | length |
| --- | --- | --- |
| Human eNOS | (Forward)5’-CCGAGTCCTCACCGCCTTCT-3’ (Reverse)5’-GGTAACATCGCCGCAGACAAA-3’ | 142 bp |
| Human CD31 | (Forward)5’-CACCTCCAGCCAACTTCACCAT-3’ (Reverse)5’-CACTGTCCGACTTTGAGGCTATCT-3’ | 90 bp |
| Human CD144 | (Forward)5’-CCAAGCCCTACCAGCCCAAAGT-3’  (Reverse)5’GCCGTGTTATCGTGATTATCCGTGA-3’ | 163 bp |


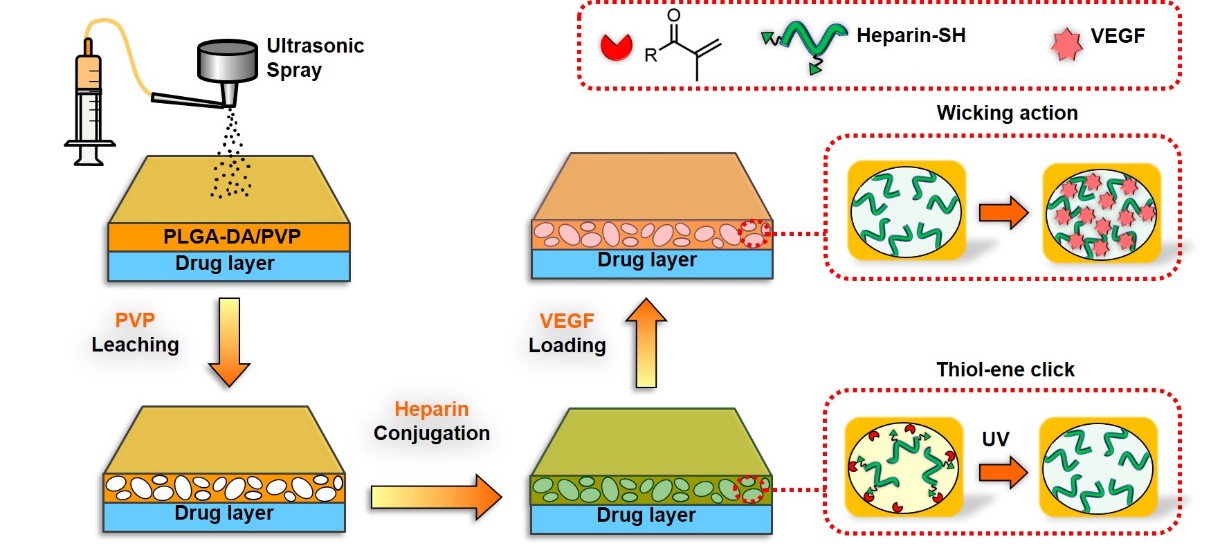


***Scheme S1.*** *Schematic illustration of the preparation of the hierarchical coating. The capillarity based wicking action realizes the spatial combination of VEGF with drugs.*


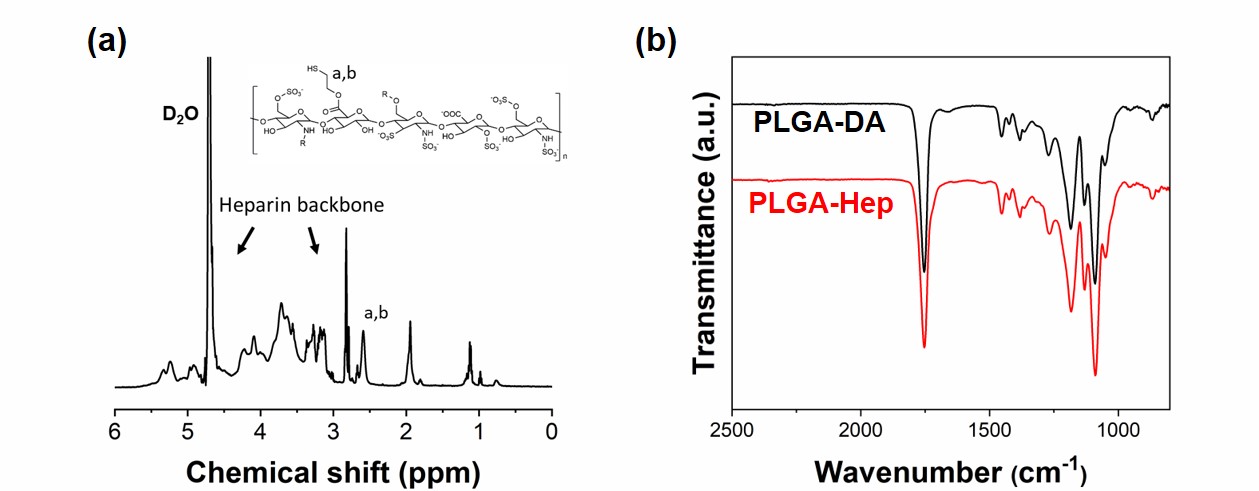


***Figure S1.*** *Heparin conjugation to the hierarchical coatings: (a) ^1^H-NMR spectrum of heparin-SH. (b) FTIR spectrum of the hierarchical coating before and after heparin conjugation, respectively. The peak decrease at 1640 cm^-1^ indicates the reaction between the methacrylate group and the thiol group.*


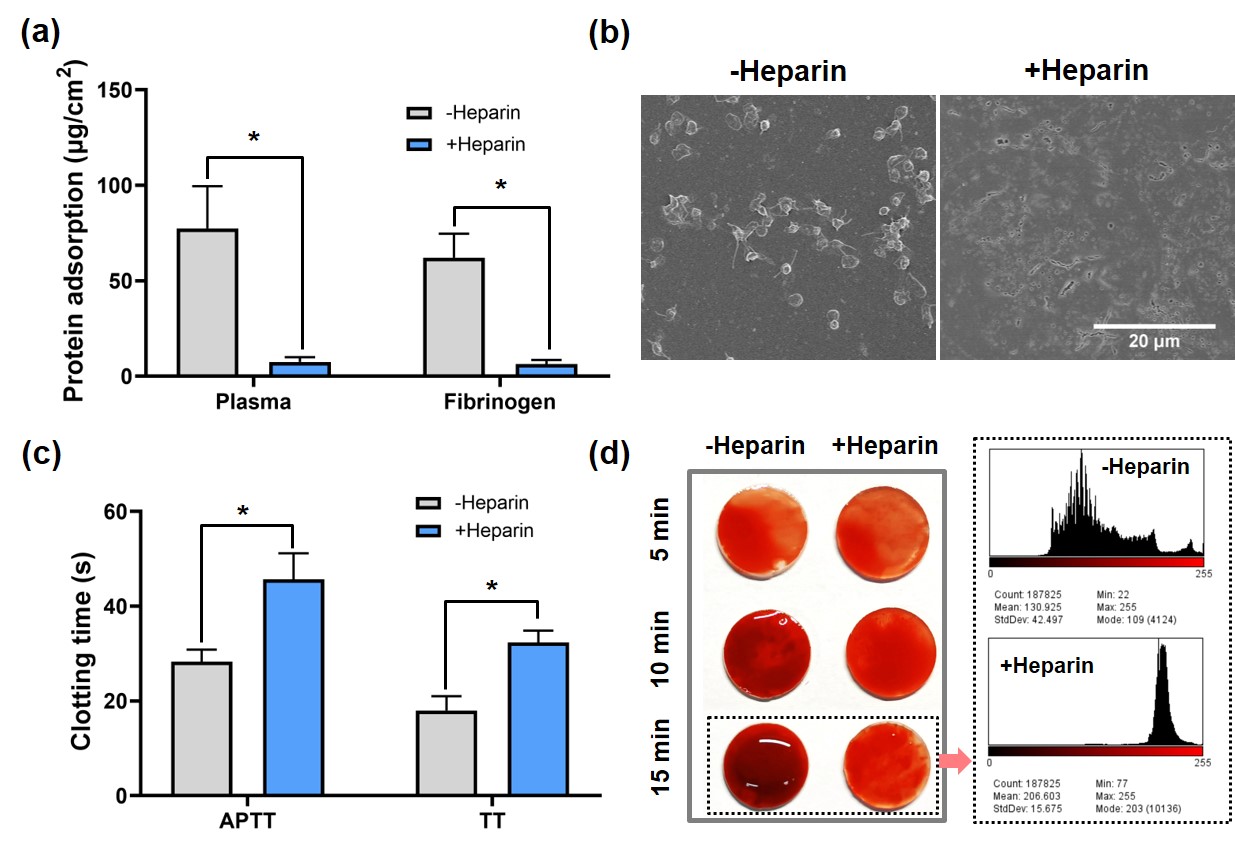


***Figure S2.*** *Blood compatibility of hierarchical coating before and after heparin conjugation: (a) Total protein adsorption by immersion in platelet-poor plasma and fibrinogen solution (1 mg/mL in PBS) for 2 h, respectively. (b) Platelet adhesion on the coatings. (c) APTT and TT tests; (d) Whole blood test at different time points. The right image shows histogram analysis of the samples of 15 min whole blood treatment. (n=3, *P < 0.05).*


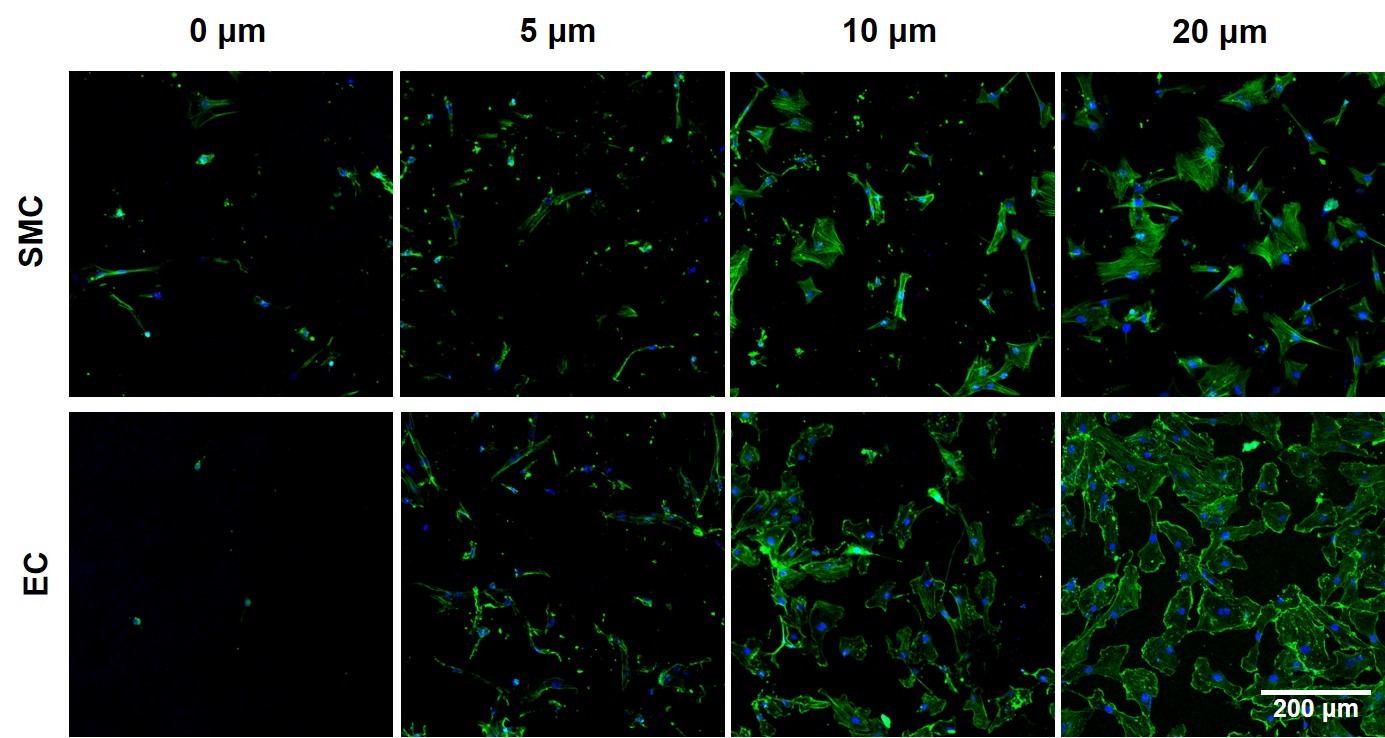


***Figure S3.*** *The adhesion and proliferation of ECs and SMCs on the hierarchical coatings with different thicknesses of the top spongy region. With the increased thickness of the top spongy layer, both ECs and SMCs showed better growth behavior.*


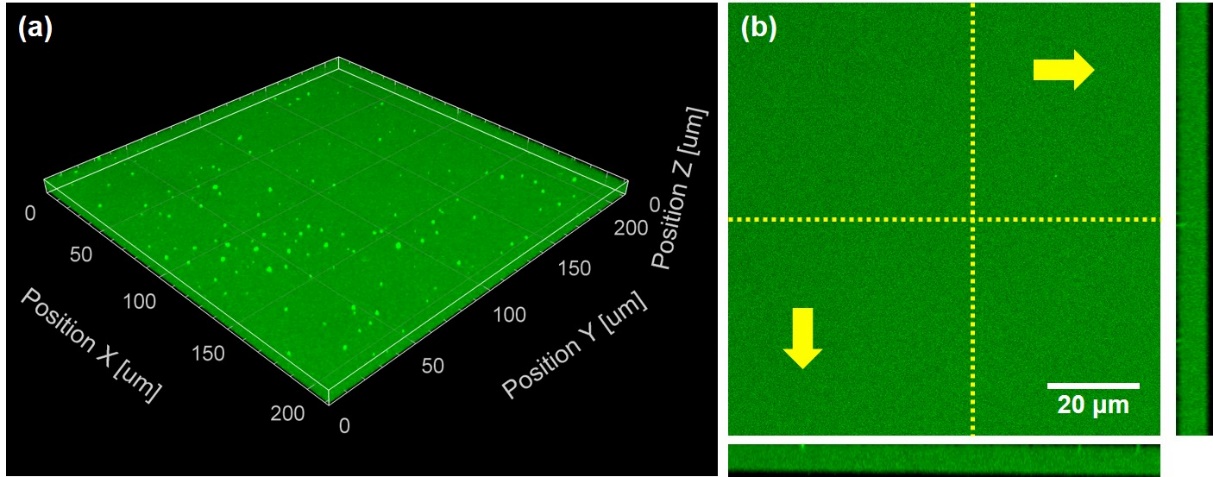


***Figure S4.*** *Confocal fluorescence micrographs with 3d view (a) and Side view (b) showing the distribution of PLL-FITC within the hierarchical coating.*

*

*

***Figure S5.*** *VEGF loading efficiency at different solution concentrations. The VEGF loading efficiency showed a similar level at different solution concentrations, indicating a reliable VEGF loading process.*


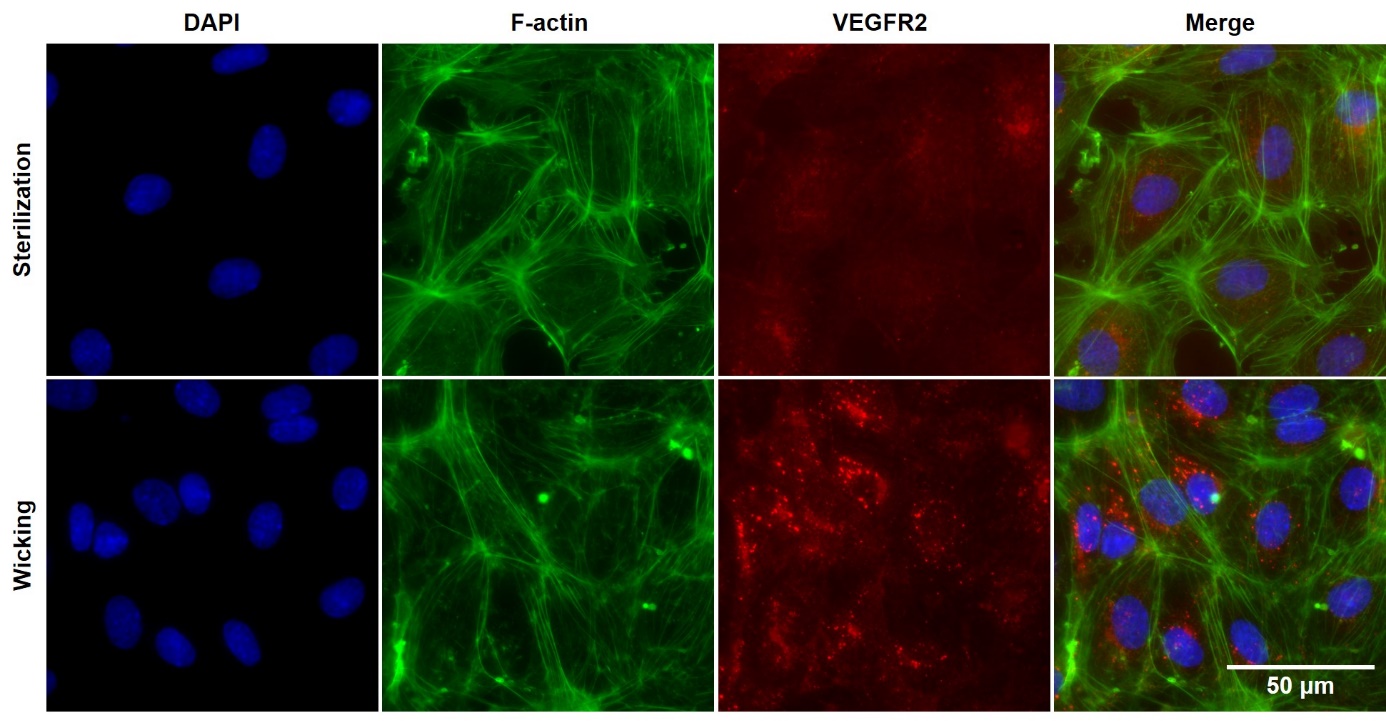


***Figure S6.*** *The internalization of VEGFR2 within ECs. The sterilized coating showed little effect on the VEGFR2 internalization, indicating the loss of bioactivity. In contrast, VEGF loaded by wicking action promoted the VEGFR2 internalization (red spot).*


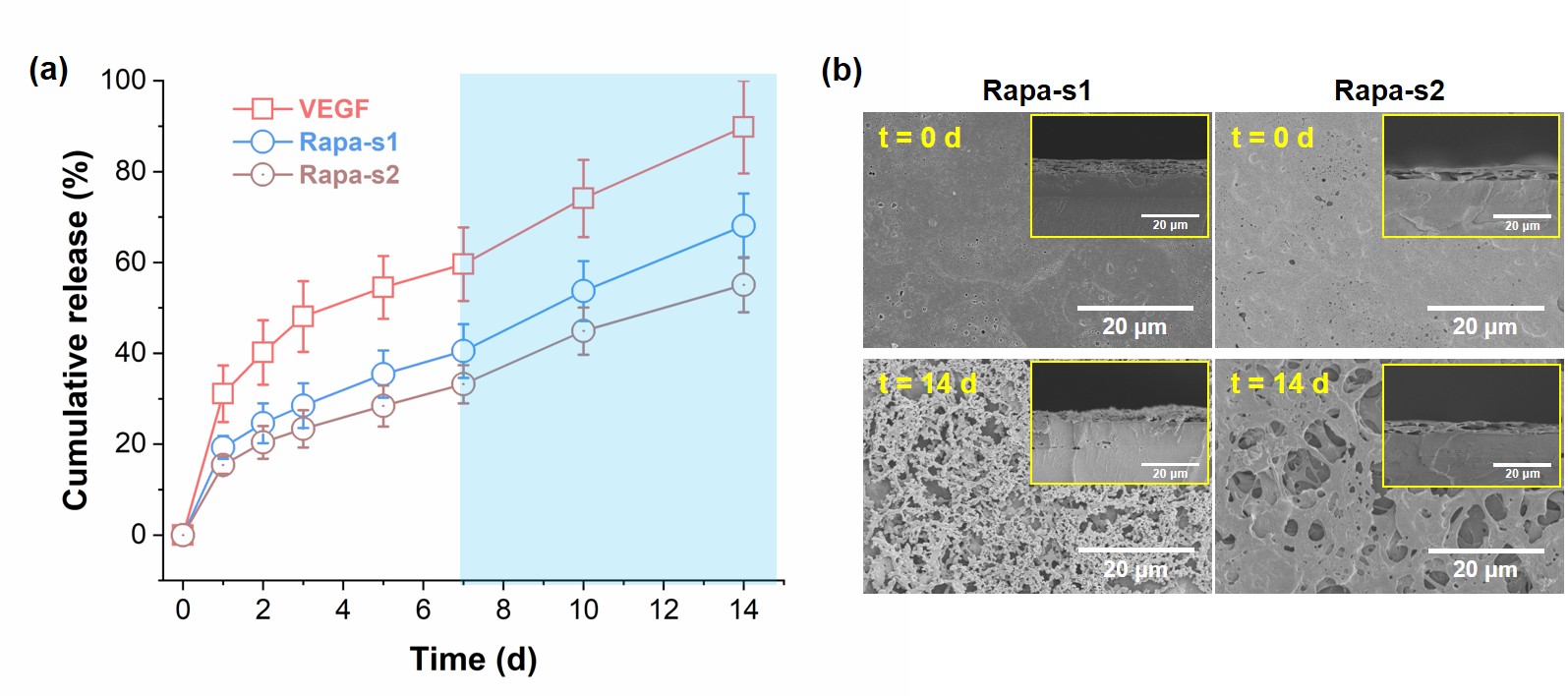


***Figure S7.*** *The lipase accelerated release behavior of hierarchical coating: (a) Cumulative release of VEGF and rapamycin in 14 d. (b) SEM micrographs of hierarchical coating before and after 14-day release. Rapa-s1: PLGA-DA/PVA~0.4; Rapa-s2: PLGA-DA/PVP~0.6.*


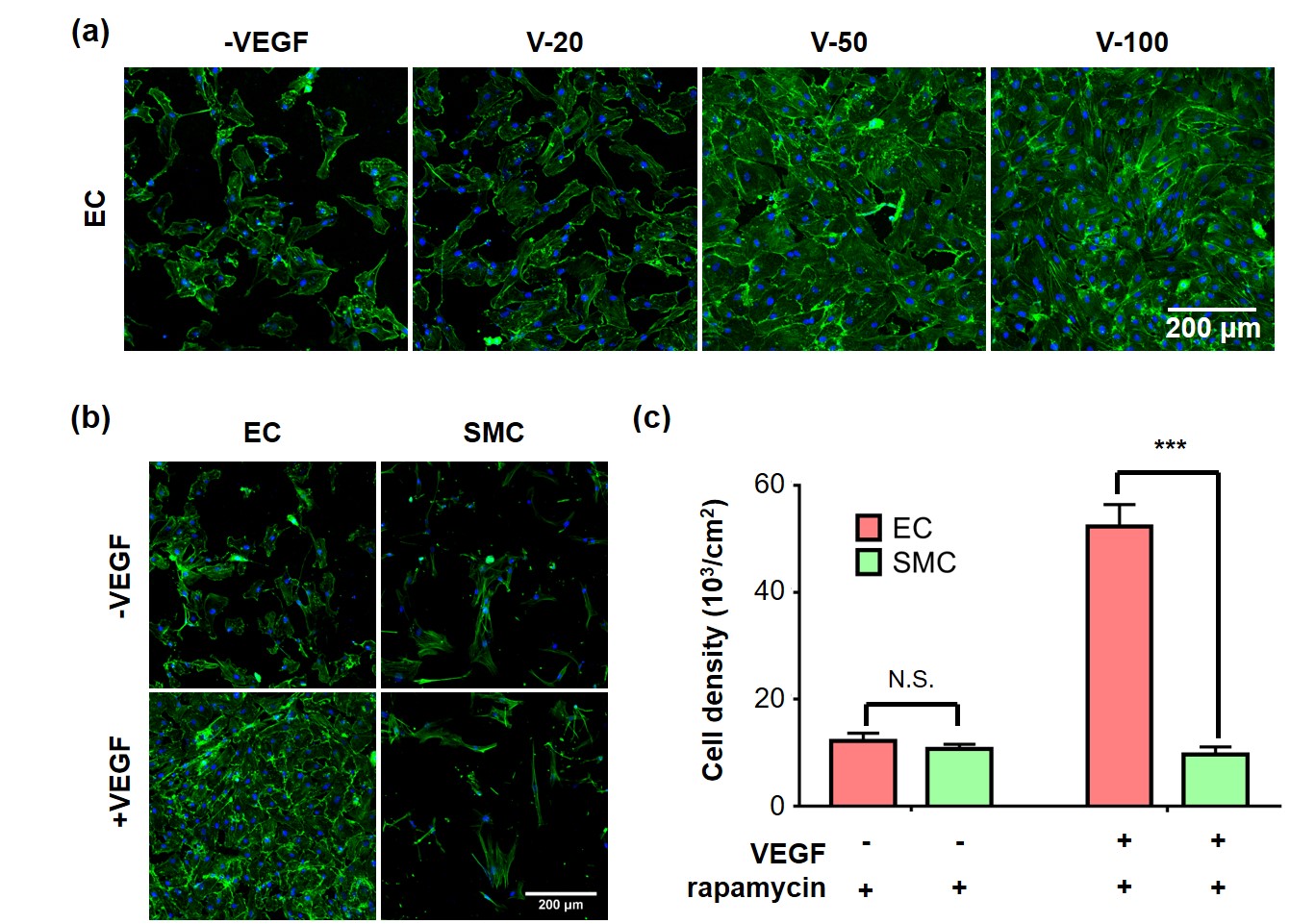


***Figure S8.*** *The cell growth behavior of ECs and SMCs on the hierarchical coatings before and after VEGF incorporation: (a) Fluorescence micrographs of ECs on the hierarchical coatings with various VEGF density. The VEGF density of V-20, V-50 and V-100 was 0.25, 0.55, and 1.01 μg/cm^2^, respectively; (b) Fluorescence micrographs of the cells (blue: DAPI; green: F-actin); (c) Corresponding cell density of the ECs and SMCs. (n=5, ***P < 0.001).*


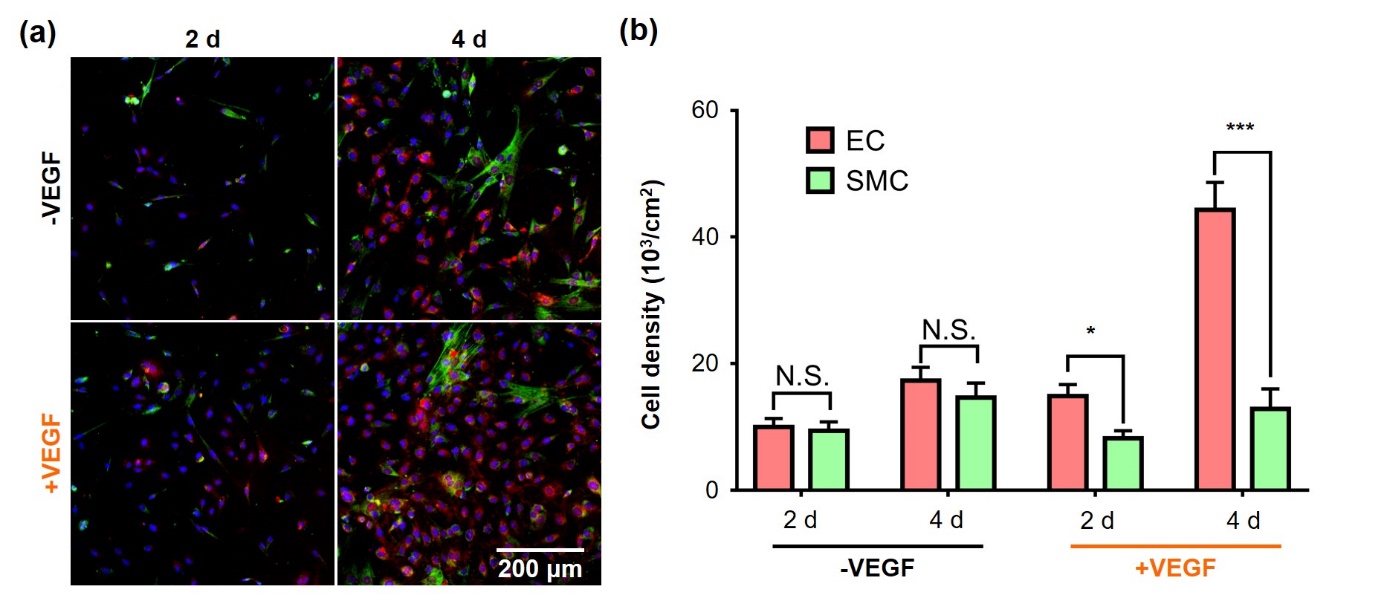


***Figure S9.*** *Co-culture of ECs with SMCs on rapamycin-free coatings. Confocal micrographs (a) and corresponding cell density of ECs and SMCs on the hierarchical coatings with/without VEGF loading, respectively. (n=5, *P < 0.05, ***P < 0.001).*


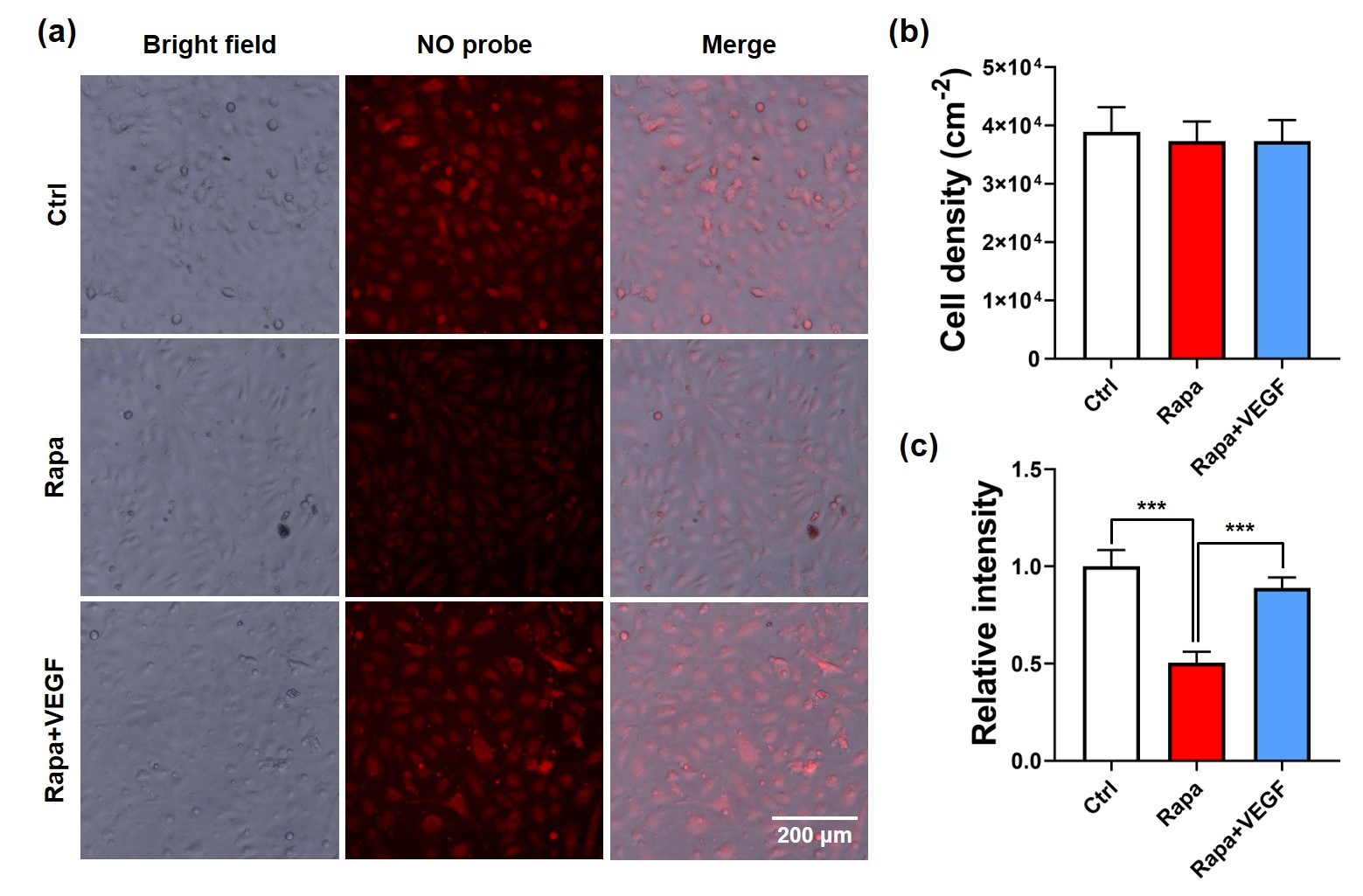


***Figure S10.*** *The nitric oxide (NO) generation of ECs: (a) Fluorescence micrographs of ECs after treatment with different samples, the red NO fluorescence probe is used to detect the NO generation. The cell density of ECs (b) and relative fluorescence intensity (c) after treatment with different samples. (the relative fluorescence intensity was normalized against control, n=5, *** p < 0.001)*


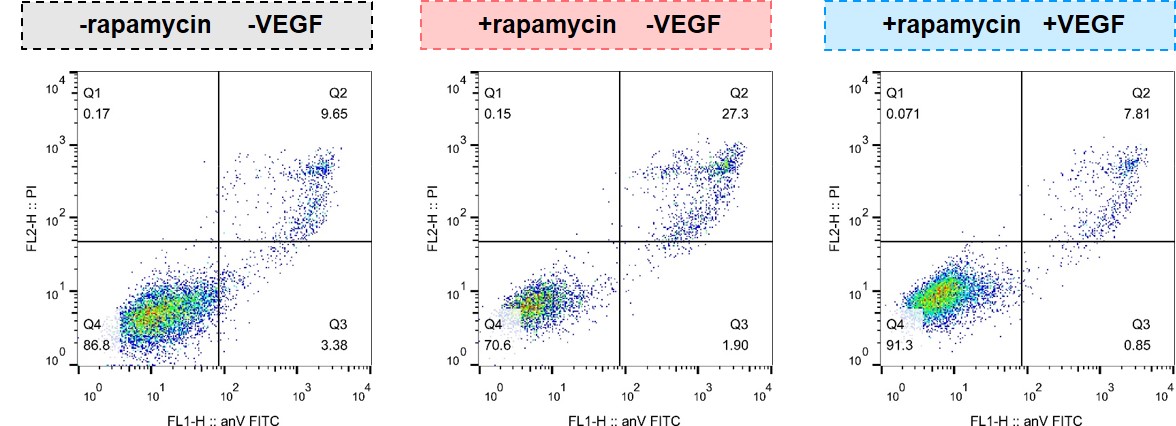


***Figure S11.*** *Evaluation of the cell apoptosis by flow cytometry. The coatings containing rapamycin alone induced substantial apoptosis of the ECs, while the spatial combination of VEGF with rapamycin dramatically enhanced cell viability.*


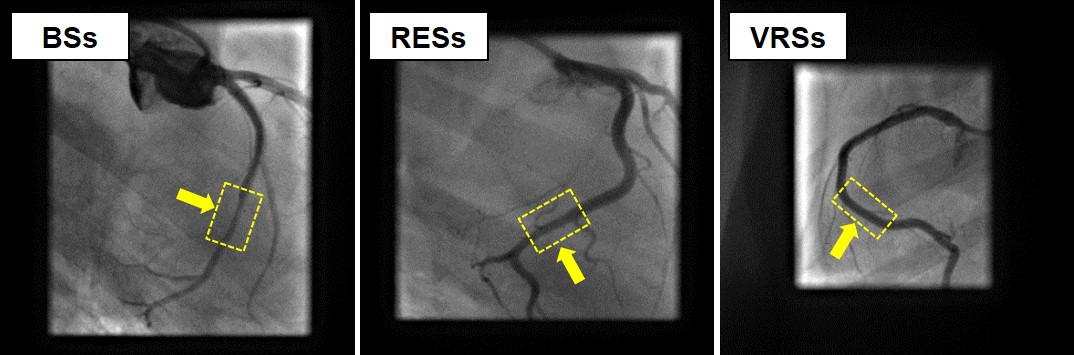


***Figure S12.*** *Coronary angiography after implantation for 6 weeks. The bare stents showed obvious in-stent restenosis, while the rapamycin coated stents and VEGF/rapamycin co-coated stents remained patency.*
